# Comparative genomics links ecological dominance and genome plasticity in sediment-derived *Vibrio diabolicus*

**DOI:** 10.3389/fmicb.2026.1813524

**Published:** 2026-04-15

**Authors:** Esmaeil ALSaleh

**Affiliations:** Department of Biological Sciences, College of Science, Sabah Al-Salem University City, Al-Shadadiya, Kuwait

**Keywords:** accessory genome, ANI, comparative genomics, core-genome phylogeny, marine sediments, pangenome, *Vibrio diabolicus*

## Abstract

Marine sediments harbor diverse *Vibrio* populations that play critical roles in benthic microbial ecology; however, the genomic determinants underlying the dominance of sediment-associated *Vibrio* taxa remain insufficiently characterized at the genome level. In this study, culture-based enumeration revealed *Vibrio diabolicus* as the dominant *Vibrio* species in marine sediment samples, providing ecological rationale for genome-resolved investigation. Because sediment-associated *vibrio* species, often responds rapidly to environmental fluctuations and organic enrichment in coastal ecosystems, their distribution and genomic characteristics may also reflect changes in sediment microbial community structure and local environmental conditions. Four sediment-derived *V. diabolicus* isolates (Vdiab_L2, Vdiab_L3, Vdiab_VA, and Vdiab_B48) were subjected to whole-genome sequencing and comparative genomic analysis alongside closely related reference genomes. Average nucleotide identity (ANI) analyses confirmed species-level assignment, with all isolates exhibiting ANI values exceeding 95% relative to *V. diabolicus* references, while remaining clearly distinct from *V. alginolyticus* and *V. parahaemolyticus* references. Core-genome phylogenetic reconstruction resolved the sediment isolates into a coherent *V. diabolicus* lineage, consistent with ANI-based relationships and demonstrating strong concordance between whole-genome similarity metrics and evolutionary history inferred from conserved genes. Pangenome analysis revealed a relatively small, conserved core genome accompanied by a dominant accessory gene pool composed primarily of shell and cloud genes, indicative of an open pangenome structure. Accessory gene clustering and presence–absence profiling further highlighted strain-specific genomic heterogeneity within the species. Importantly, this study focuses specifically on comparative genomic structure rather than comparative functional genomics, aiming to establish a genome-level evolutionary framework for sediment-associated *V. diabolicus* populations. Together, these findings demonstrate that *V. diabolicus* combines ecological dominance in marine sediments with extensive genomic plasticity, a combination likely facilitating persistence and adaptation within heterogeneous benthic environments. This study provides a comprehensive comparative genomic baseline for understanding sediment-associated *V. diabolicus* populations and establishes a framework for future ecological and functional genomic investigations.

## 1 Introduction

Marine sediments represent complex and dynamic ecosystems that harbor highly diverse microbial communities and play a central role in biogeochemical cycling, organic matter turnover, and nutrient regeneration in coastal and shelf environments. Among sediment-associated bacteria, members of the genus *Vibrio* are particularly prominent due to their metabolic versatility, rapid growth, and capacity to adapt to fluctuating physiochemical conditions (Thompson et al., 2004; Hunt et al., 2008). While many *Vibrio* species are well known for their roles as pathogens or symbionts, an increasing body of evidence highlights their ecological importance as free-living and biofilm-forming bacteria in marine sediments (Polz et al., 2013).

Advances in whole-genome sequencing have fundamentally transformed the taxonomy and ecology of *Vibrio*, revealing extensive genomic diversity, frequent horizontal gene transfer, and complex population structures molded by environmental selection (Thompson et al., 2005; Shapiro et al., 2012). Genome-resolved analyses have demonstrated that many *Vibrio* species possess open pangenomes, in which a relatively small, conserved core genome is accompanied by a large and dynamic accessory gene pool (Tettelin et al., 2008; Lukjancenko et al., 2010). This genomic organization is thought to emphasize the outstanding ecological flexibility of *Vibrio* species allowing rapid adaptation to distinct niches such as the water column, host-associated environments, and benthic sediments.

Recent genomic studies have further emphasized the importance of comparative genomics for understanding the ecological diversification of *Vibrio* species. In particular, analyses of *Vibrio diabolicus* genomes have revealed considerable genetic diversity and the presence of extensive accessory gene repertoires. Comparative genomic investigation of *V. diabolicus* and related taxa demonstrated that this species possesses a large accessory genome relative to the conserved core gene set, highlighting the role of genome plasticity in shaping evolutionary diversification within the species complex (Turner et al., 2018). These findings support the concept that genomic flexibility plays a central role in enabling *Vibrio* species to colonize heterogenous marine environments.

In addition to comparative genomic investigations, several studies have characterized the genomic architecture and metabolic potential of *V. diabolicus*. Genome sequencing of the species identified biosynthetic gene clusters involved in the production of the exopolysaccharide HE800, revealing genetic features associated with ecological interactions and environmental persistence (Goudenege et al., 2014). Environmental studies have further deocumented the occurrence of *V. diabolicus* in estuarine and coastal ecosystems, where genomic analyses identifies diverse gene categories within the core and accessory genomes of environmental strains (Dias, 2020). Isolation and characterization of *V. diabolicus* from aquaculture environments have also demonstrated the presence of distinct genetic lineages identifies through molecular and genomic approaches, emphasizing the ecological distribution and genomic diversity of this species (Song et al., 2021).

A defining characteristic of *Vibrio* species is their highly flexible genome architecture, which is typically organized as an open pangenome. In such systens, the number of accessory genes continues to increase as additional genomes are incorporated into comparative analyses, reflecting extensive horizontal gene transfer and genomic recombination within the lineage (Tettelin et al., 2008; Shapiro et al., 2012; Polz et al., 2013). This accessory genome often encodes niche-specific traits that contribute to ecological adaptation and environmental specialization. Similar patterns have been observed in other *Vibrio* species, including pangenome analyses of *Vibrio mimicus*, which revealed substantial variability in accessory gene content among closely related strains (Guardiola-Avila et al., 2021). These findings highlight the importance of genome-scale comparative analyses for understanding how genetic diversity contributes to ecological success within Vibrionacae.

Among sediment-associated *Vibrio* species, *Vibrio diabolicus* has received comparatively limited attention despite repeated isolation from marine sediments and biofilms. Originally described from deep-sea hydrothermal vent environments, *V. diabolicus* has since been reported from diverse marine habitats, suggesting a broad ecological distribution and adaptive capacity. However, the genomic basis underlying its persistence and dominance in sedimentary environments remains poorly characterized. In particular, few studies have directly linked quantitative ecological dominance with genome-resolved comparative analyses to evaluate how genome structure and gene content contribute to sediment-associated success.

Accurate species allocation within the genus *Vibrio* has historically been challenging due to high phenotypic similarity and extensive genetic exchange among closely related taxa. Genome-based metrics such as average nucleotide identity (ANI) have therefore become essential tools for robust taxonomic assignment, with a threshold of approximately 95% ANI widely accepted for species-level discrimination (Goris et al., 2007; Richter and Rosselló-Móra, 2009). When combined with core-genome phylogenetic reconstruction, ANI provides a powerful framework for resolving evolutionary relationships and validating species identity within genomically complex bacterial groups such as *Vibrio*.

Beyond species confirmation, comparative genomics offers critical insights into the relative contributions of core and accessory genomes to ecological differentiation. Core genes encode essential cellular functions and tend to be evolutionarily conserved, whereas accessory genes—often acquired through horizontal gene transfer—frequently encode niche-specific traits related to nutrient acquisition, stress tolerance, and environmental interactions (Koonin and Wolf, 2008; Polz et al., 2013). In marine bacteria, accessory genome variation has been shown to drive fine-scale population structure and local adaptation, particularly in heterogenous environments such as sediments.

In this study, culture-based enumeration was combined with genome-resolved comparative analyses to investigate sediment-associated *Vibrio diabolicus* populations. First, quantitative enumeration was used to access the relative abundance of *Vibrio* species in marine sediment samples, identifying *V*. *diabolicus* as the dominant taxon. Subsequently, four sediment-derived *V*. *diabolicus* isolates were subjected to whole-genome sequencing and analyzed alongside closely related reference genomes. Using ANI, core-genome phylogenetics, and pangenome analysis, we aimed to (i) confirm species-level identity of sediment isolates, (ii) resolve their evolutionary relationships, and (iii) characterize the structure and diversity of the *V*. *diabolicus* pangenome. Importantly, the present study focuses specifically on comparative genomic structure rather than comparative functional genomics, emphasizing genome architecture, evolutionary relationships, and pangenome composition among sediment-derived *V. diabolicus* isolates. By explicitly integrating ecological dominance with comparative genomic structure, this work provides a comprehensive framework for understanding how genomic structure supports the success of *V*. *diabolicus* in marine sediments. The findings contribute to broader efforts to link microbial genome evolution with ecological function in benthic marine ecosystems and establish a baseline for future functional genomics investigations targeting sediment-adapted *Vibrio* populations.

## 2 Materials and methods

### 2.1 Study area and sediment sampling

Surface coastal sea sediments were collected from a nearshore site in Kuwait (29.37975° N, 47.85843° E) in September. Sampling was performed in shallow water (~5 cm depth), and the surface water temperature at the time of collection was 28.3 °C, measured using a standard glass thermometer. After removing surface small stones and pebbles, sediments samples were aseptically collected from six concentric sampling points spaced 25 m apart, transferred into sterile containers, transported to the laboratory on ice, and processed immediately upon arrival. For culture-based enumeration and bacterial isolation, sediment subsamples were pooled and homogenized prior to serial dilution and plating.

### 2.2 Isolation, enumeration of bacterial groups, and identification of *Vibrio* species

Culturable bacterial abundance in sediment samples was quantified using standard plate count methods. Serial dilutions and preparation of sediment suspensions were performed following (International Organization for Standardization 2017) while enumeration of culturable microorganisms was conducted according to (International Organization for Standardization 2018). Briefly, approximately 10 g (wet weight) of sediment was suspended in sterile saline and homogenized by vortexing. The suspensions were serially diluted tenfold and aliquots (100 μl) of appropriate dilutions were spread-plated on selective and non-selective media. Nutrient agar (NA) was used to enumerate total heterotrophic bacteria and to estimate the relative abundance of Gram-negative and Gram-positive populations merely by applying Gram stain to grown bacterial colonies. The thiosulfate-citrate-bile salts-sucrose (TCBS) agar was used for selective enumeration of *Vibrio spp*. and preliminary differentiation of *Vibrio* species.

Plates were incubated under aerobic conditions at 25 °C for 24 h (NA) or up to 48 h (TCBS). Colonies were counted from plates yielding countable ranges, and bacterial abundance was expressed as colony-forming units per gram of sediment (CFU g^−1^). For each bacterial group, replicate plating was performed (*n* = 5), and quantitative results were summarized as mean ± standard error of the mean (SEM). Enumeration procedures were consistent with internationally accepted guidelines for culture-based microbial analysis of environmental samples, including APHA Standard Methods for the Examination of Water and Wastewater (23rd edition) and relevant ISO standards (e.g., International Organization for Standardization, 2018). These methods ensured methodological reproducibility and comparability with previous sediment microbiology studies.

Enumeration data was used to assess the relative abundance of *Vibrio* species in all sediment samples and to identify *Vibrio diabolicus* as the dominant taxon selected for subsequent genome-resolved analyses. Representative colonies were purified by repeated streaking and subjected to preliminary phenotypic and biochemical characterization. Identification criteria included colony morphology, swarming behavior, colony color on TCBS agar, growth temperature range, sucrose fermentation, Voges-Proskauer reaction, indole production, ornithine decarboxylase activity, citrate utilization, sensitivity to O/129 (159 μg), and hemolytic activity. The results of phenotypic and biochemical tests were interpreted according to standard criteria described in recognized microbiological identification manuals, including (Bergey's Manual Trust 2015) and the Manual of Clinical Microbiology (12th edition) by (Carroll et al. 2019). Bacterial identities were further confirmed by 16s RNA sequencing. For this purpose, bacterial genomic DNA was extracted using the Wizard Genomic DNA purification kit (Promega, Madison, WI, USA) according to the manufacturer's protocol. Partial 16S rRNA gene sequences were amplified using primers 785F and 907R under standard PCR conditions. Amplicons were purified and sequenced using ABI 3730xl DNA Analyzer (Applied Biosystems, Foster City, CA, USA). Consensus sequences were compared against the NCBI nucleotide database using BLASTn. Closest phylogenetic affiliations were determined based on ≥98% sequence similarity to accurately described type strains (Al-Saleh et al., 2009; Al-Saleh and Akbar, 2015).

### 2.3 Whole-genome sequencing and data processing

Pure cultures of four sediment-derived *Vibrio diabolicus* isolates (Vdiab_L2, Vdiab_L3, Vdiab_VA, and Vdiab_B48) were grown in nutrient broth under aerobic conditions. Cells were harvested by centrifugation and genomic DNA was extracted using GenElute Bacterial Genomic DNA Kit (Macrogen Inc., Daejeon, South Korea) according to the manufacturer's instructions. DNA concentration and purity were assessed spectrophotometrically, while DNA integrity was verified by agarose gel electrophoresis. Sequencing libraries were constructed from purified genomic DNA using random fragmentation, followed by ligation of platform-specific adapters at both the 5′ and 3′ ends. Adapter-lighted fragments were PCR-amplified and purified prior to sequencing. Whole-genome sequencing was performed using Illumina sequencing-by-synthesis (SBS) technology (Macrogen Inc., Daejeon, South Korea). Prepared libraries were loaded onto flow cells, where DNA fragments hybridized to surface-bound oligonucleotides and underwent clonal amplification through bridge amplification to form discrete clusters. Sequencing was conducted using reversible terminator chemistry. Raw sequencing data were generated in FASTQ format for subsequent analyses. Raw paired-ends reads were subjected to initial quality assessment to evaluate sequencing performance, including total read number, total bases generated, GC content, and base-quality distributions. Per-base and per-cycle quality metrics were examined to ensure overall data reliability. Adapter removal and quality filtering were performed using Trimmomatic (Version 0.36.6), eliminating residual adapter sequences and low-quality bases to reduce analytical bias. After preprocessing, filtered reads were reassessed to calculate updated statistics, including retained read counts, total bases, GC percentage, and the proportions of bases with Phred quality scores ≥20 (Q20) and ≥30 (Q30). Only high-quality filtered reads were retained for genome assembly. Accordingly, using Galaxy bioinformatics platform for genome assembly and comparative genomic analyses, high quality reads were assembled *de novo* using SPAdes (Version 4.2.0), which employs a de Bruijn graph-based algorithm optimized for bacterial genomes (Bankevich et al., 2012; Antipov et al., 2016). Assemblies were generated using default bacterial parameters and resulting contigs were evaluated for completeness and quality using standard assembly metrics, including total assembly size, number of contigs, and N50 values. The final assemblies were used for downstream annotation and comparative-genomic analyses. Assembled genomes were annotated using Prokka (Version 1.14.6), which predicts protein-coding sequences, ribosomal RNA genes, transfer RNA genes, and other genomic features using curated reference databases (Seemann, 2014). Annotated genome files served as the basis for average nucleotide identity (ANI) estimation pangenome construction, and core-genome phylogenetic analyses. The primary objective was species-level comparative genomics and pangenome characterization. Accordingly, all downstream analyses were performed using assembled and annotated genomes.

### 2.4 Reference genome selection

Nine reference genomes were retrieved from the NCBI database, including five *Vibrio diabolicus* reference genomes, three *Vibrio alginolyticus* genomes, and one *Vibrio parahaemolyticus* genome used as an outgroup. Reference genomes were selected based on completeness, annotation quality, and taxonomic relevance.

### 2.5 Average nucleotide identity (ANI) analysis

Pairwise average nucleotide identity (ANI) was calculated using FastANI (Version 1.3), implemented in Galaxy (Jain et al., 2018). ANI values were computed for all combinations of sediment isolates and reference genomes. A threshold of 95% ANI was used to confirm species-level relatedness. ANI results were visualized as a heatmap to assess genomic similarity patterns.

### 2.6 Core-genome alignment and phylogenetic analysis

Core genes shared among all genomes were identified using Roary (Version 3.13.0) with a BLASTp identity cutoff of 95% (Page et al., 2015). Concatenated core-gene alignments were generated and used to construct a maximum-likelihood phylogenetic tree with IQ-TREE (Version 2.4.0). The best-fit substitution model was automatically selected, and branch support was assessed using standard likelihood-based approaches (Nguyen et al., 2015). The phylogeny was rooted using *Vibrio parahaemolyticus* as an outgroup.

### 2.7 Pangenome analysis

Pangenome analysis was conducted using Roary to classify genes into core, soft-core, shell, and cloud categories based on their distribution across genomes. Gene counts and relative proportions were calculated to characterize the pangenome structure and to assess genomic diversity within *Vibrio diabolicus*.

### 2.8 Accessory genome clustering and gene presence-absence analysis

Accessory gene content was examined using the Roary binary gene presence-absence matrix. Hierarchical clustering based on accessory gene profiles was performed to visualize relationships driven by the flexible genome. Additionally, heatmap visualization of variable accessory genes was generated to highlight strain-specific gene repertoires.

### 2.9 Correlation between ANI and core-genome phylogenetic distance

To evaluate concordance between whole-genome similarity and evolutionary relationships, ANI values were compared with core-genome phylogenetic distances derived from the maximum-likelihood tree. Scatter plot analysis was used to assess the relationship between ANI and phylogenetic divergence.

### 2.10 Data visualization

All figures were generated using Python-based visualization libraries and customized for publication-quality resolution. Heatmap, phylogenetic trees, and comparative plots were formatted to ensure consistency in all figures and compatibility with journal submission requirements.

### 2.11 Statistical analysis

Descriptive statistics were used to summarize enumeration and genomic data. Comparative analyses focused on relative differences in gene content, ANI values, and phylogenetic relationships rather than hypothesis-driven statistical testing, consistent with standard practices in comparative genomics.

## 3 Results

### 3.1 Enumeration and dominance of *Vibrio diabolicus* in marine sediments

Culture-based enumeration revealed clear dominance of *Vibrio diabolicus* among recovered *Vibrio* species from marine sediment samples (Table 1). In all sampling sites, *V*. *diabolicus* exhibited the highest colony-forming units (CFU g^−1^ dry sediment), consistently exceeding other *Vibrio* species. Consistent with this pattern, mean counts for *V. diabolicus* were 5.31 × 10^5^ CFU g^−1^ compared to 1.56 × 10^5^ CFU g^−1^ for *V. alginolyticus* (Table 1). A one-way ANOVA performed on log10-transformed CFU g^−1^ values (*n* = 5 per species) confirmed that *V. diabolicus* abundance was significantly higher than *V*.*alginolyticusF*_(1, 8)_ = 1.13 × 10^10^, *p* < 0.001. This quantitative dominance provided the ecological rationale for selecting *V*. *diabolicus* as the pivotal taxon for comparative genomic analysis.

**Table 1:** Enumeration of culturable bacterial groups and dominance of *Vibrio diabolicus*.

No.	Bacterial group	Bacterial count (CFU g^−1^ sea sediment)
1	Total heterotrophic bacteria	3.65 × 10^6^ ± 2.4
2	Total Gram-negative bacteria	2.30 × 10^6^ ± 2.2
3	Total *Vibrio* spp.	7.80 × 10^5^ ± 2.4
4	*Vibrio diabolicus*	5.31 × 10^5^ ± 1.8
5	*Vibrio alginolyticus*	1.56 × 10^5^ ± 1.7
6	*Vibrio parahaemolyticus*	9.38 × 10^4^ ± 1.2

Culture-based enumeration of total heterotrophic bacteria, total *Vibrio* spp., and individual *Vibrio* species recovered from marine sediment samples. Values are expressed as colony-forming units per gram of dry sediment (CFU g^−1^) and represent mean ± SEM based on five independent plating replicates. Enumeration revealed *Vibrio diabolicus* as the numerically dominant *Vibrio* species in all sampling sites, providing the ecological rationale for its selection as the pivotal taxon for genome-resolved comparative analysis. Values represent the mean of five independent replicates ± standard error of the mean (SEM).

### 3.2 Genome assembly quality and general genomic features

Whole-genome sequencing and assembly of the four sediment-derived *V. diabolicus* isolates generated high-quality draft genomes ranging from 4.93 Mb to 5.32 Mb (Table 2). Genome sizes were highly similar among isolates, with L2 and L3 showing nearly identical assemblies (4.93–4.94 Mb), while VA exhibited the largest genome (5.32 Mb) and B48 an intermediate size (5.13 Mb). Assembly fragmentation varies between 53 and 105 contigs, with L2 displaying the most contiguous assembly (53 contigs) and L3 and VA showing higher contigs counts (105 and 103, respectively). Genome annotation predicted 4,406–4,841 protein-coding sequences (CDS) in the isolates, with the highest number detected in VA, consistent with its larger genome size. Ribosomal RNA gene counts ranged from 3 to 5, while tRNA genes varied between 67 and 77 among genomes. Overall, the assemblies show comparable genome sizes and coding capacities in the isolates, indicating consistent genomic features within the anlayzed *V. diabolicus* strains and supporting species-level genomic coherence.

**Table 2:** Genome assembly and annotation statistics of sediment-derived *V. diabolicus*.

Isolate	Assembly size (bp)	No. contigs/scaffolds	Protein-coding genes (CDS)	rRNA genes	tRNA genes
L2	4,932,909	53	4,406	4	77
L3	4,942,954	105	4,406	4	76
VA	5,324,656	103	4,841	3	72
B48	5,131,608	78	~4,600	5	67

Whole-genome sequencing, assembly, and annotation statistics for sediment-derived *Vibrio diabolicus* isolates (Vdiab_L2, Vdiab_L3, Vdiab_VA, and Vdiab_B48). Parameters include genome size, GC content, number of contigs, N50, predicted coding sequences (CDSs), and RNA features. All assemblies met quality thresholds suitable for downstream comparative genomic and phylogenomic analyses. Genome assemblies were generated using SPAdes and annotated using Prokka. Assembly size, contig/scaffold counts, and annotation features were extracted from Prokka summary and log files generated in Galaxy.

### 3.3 Average nucleotide identity confirms species-level assignment

Pairwise ANI analysis demonstrated that all four sediment isolates shared ANI values well above the accepted species threshold of 95% when compared to *V*. *diabolicus* reference genomes (Table 3). In contrast, ANI values relative to *V*. *alginolyticus* reference genomes were substantially lower, confirming taxonomic distinction.

**Table 3:** Pairwise ANI values among *V. diabolicus* isolates and reference genomes.

Comparison of isolate vs. reference genome	ANI range (%)	Taxonomic interpretation
*V. diabolicus* isolates vs. *V. diabolicus*	97.0–99.2	Same species
*V. diabolicus* isolates vs. *V. alginolyticus*	90.5–92.3	Distinct species
*V. diabolicus* isolates vs. *V. parahaemolyticus*	< 90.0	Distantly related

Summary of average nucleotide identity (ANI) values among sediment-derived *Vibrio diabolicus* isolates and reference genomes. Values represent pairwise genome comparisons. Consistently high ANI values among *V. diabolicus* gnomes support their classification within a single species and demonstrate limited core-genome divergence despite ecological and geographic separation.

Pairwise ANI values were calculated using FastANI. Species delineation thresholds follow established genomic criteria (≥95%−96% ANI for same species).

### 3.4 Core-genome phylogeny resolves evolutionary relationships

**Figure 1:**
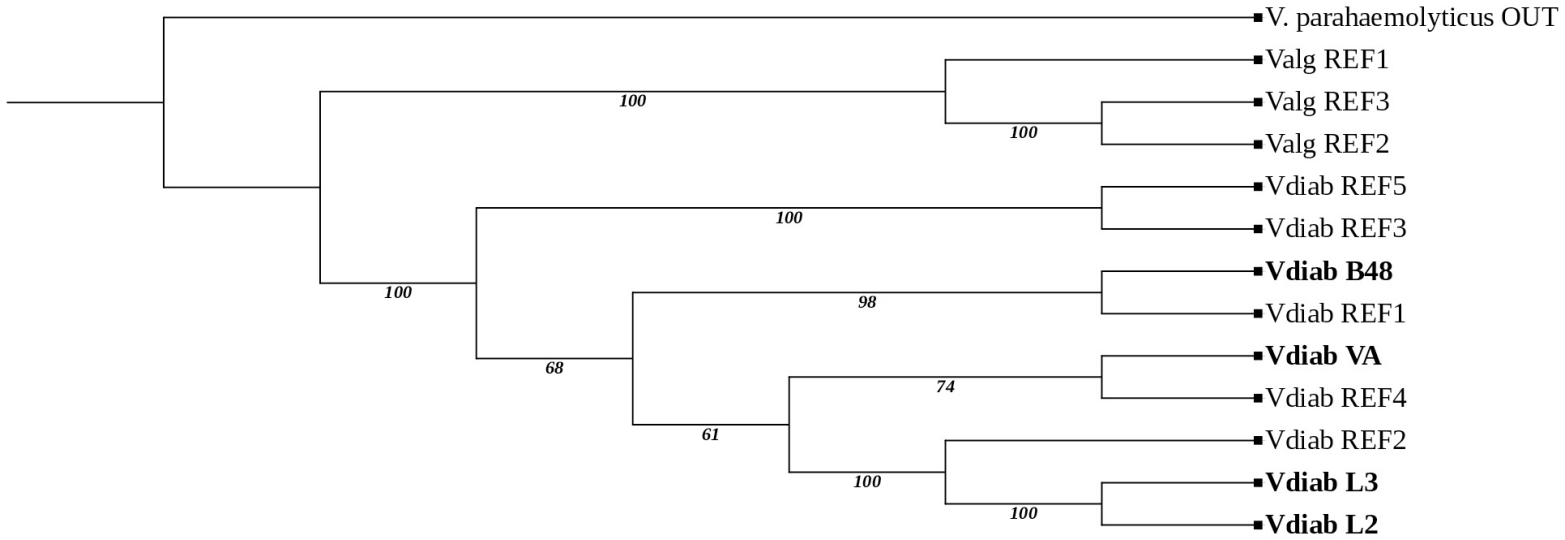
Core-genome phylogeny and strain-level genomic differentiation among *Vibrio* genomes. Maximum-likelihood inferred from a concatenated alignment of single-copy core genes using IQ-TREE under the best-fit substitution model GTR+F+I+R7. Node labels indicate SH-aLRT support (%) and ultrafast bootstrap support (%). Sediment-derived *Vibrio diabolicus* isolates (L2, L3, VA, and B48; shown in bold) cluster robustly within the *V. diabolicus clade* and are clearly separated from *V. alginolyticus* reference genomes. The tree is rooted with *Vibrio parahaemolytics* as an outgroup. The phylogenetic structure is consistent with accessory gene presence-absence clustering, highlighting strain-level genomic differentiation among sediment isolates and supporting their placement within distinct lineages of *V. diabolicus*.

Maximum-likelihood phylogenetic reconstruction based on concatenated core genes clustered all sediment isolates within a well-supported *V*. *diabolicus* clause (Figure 1). The maximum-likelihood tree inferred using IQ-TREE under the best-fit substitution model GTR+F+I+R7 showed strong statistical support across most internal nodes, with SH-aLRT and ultrafast bootstrap values generally exceeding 95%. The sediment-derived isolates L2, L3, VA, and B48 formed a well-supported cluster within the *V. diabolicus* clade, confirming their taxonomic placement and close genomic relatedness to reference *V. diabolicus* genomes. Within this clade, isolates L2 and L3 grouped together with extremely short branch lengths, indicating near-identical genomic composition, whereas VA and B48 occupied distinct but closely related sublineages. In contrast, *V. alginolyticus* reference genomes formed a separate clade clearly distinct from *V. diabolicus*. The tree was rooted with *V. parahaemolyticus* providing a stable phylogenetic framework. The topology was consistent with accessory gene clustering, ANI-based similarity patterns supporting strain-level genomic differentiation among sediment isolates.

### 3.5 Pangenome composition reveals an open genome structure

Roary pangenome analysis identified a relatively small core genome shared across all analyzed genomes, while shell and cloud genes constituted the majority of the pangenome (Table 4). The predominance of cloud genes indicates extensive strain-specific gene contents and ongoing genome diversification, consistent with an open pangenome structure.

**Table 4:** Overall pangenome size of sediment-derived *V. diabolicus* isolates and all reference genomes based on Roary.

Category	Gene clusters (*n*)	% of total pangenome (*n* = 9,374)
Core	3,746	40.0%
Soft-core	0	0.0%
Shell	1,914	20.4%
Cloud	3,714	39.6%
**Total**	**9,374**	**100%**

Summary statistics of the *Vibrio diabolicus* pangenome derived from sediment isolates and reference genomes. The table reports the total number of orthologous gene clusters and their portioning into core genes (present in all genomes), shell genes (shared by multiple but not all genomes), and cloud genes (rare or strain-specific). This distribution reflects an open-pangenome structure characteristic of environmentally distributed *Vibrio* species. The bold values indicate the total number of orthologous gene clusters assigned by Roary which by definition represents 100% of the pangenome.

### 3.6 Accessory gene clustering and presence-absence patterns

**Figure 2:**
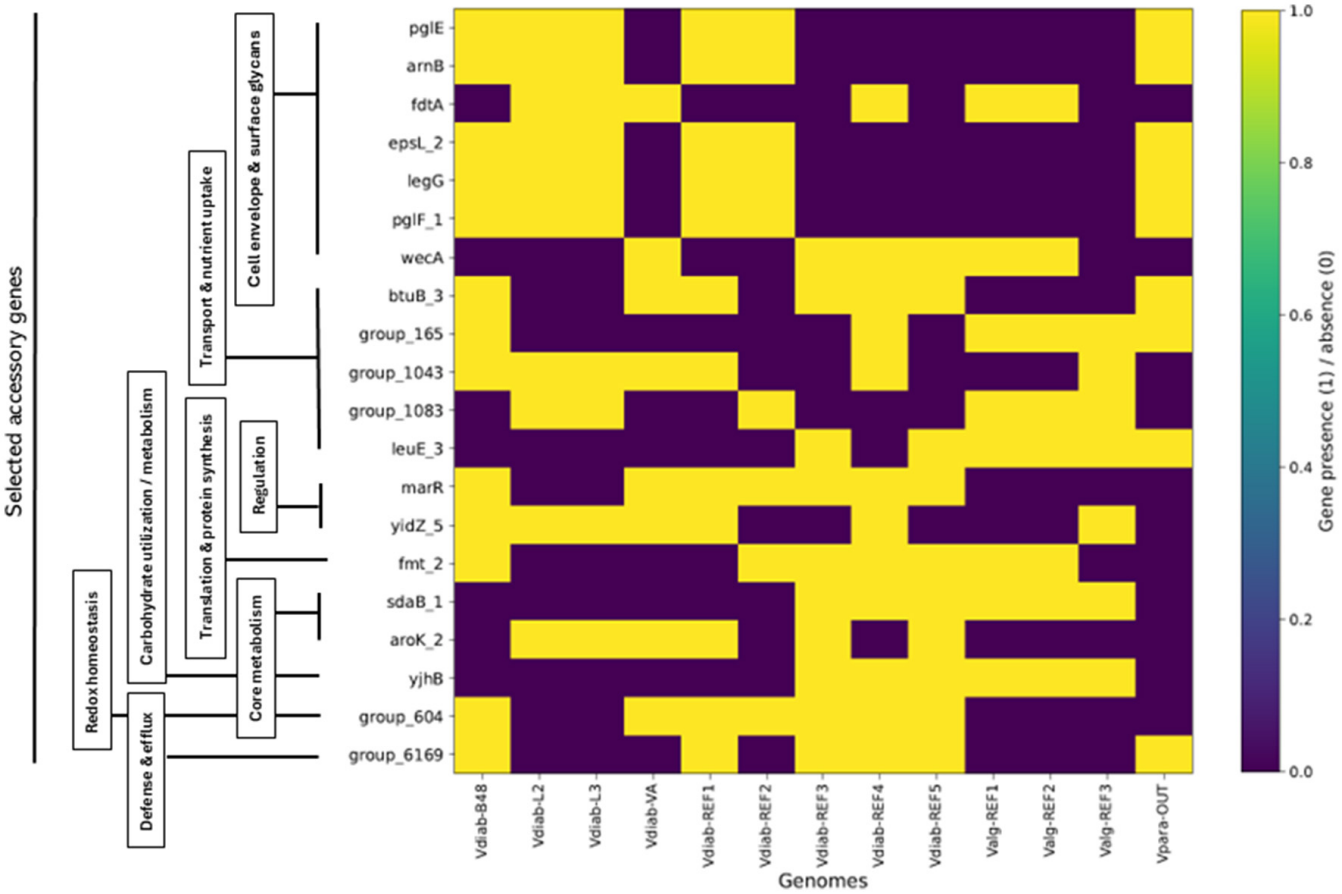
Functional organization of selected accessory genes of *Vibrio* genomes. Heatmap showing the binary presence-absence patterns of top 20 selected accessory genes identified from the Roary pangenome matrix for 13 genomes: four sediment-derived *Vibrio diabolicus* isolates (Vdiab_L2, Vdiab_L3, Vdiab_VA, and Vdiab_B48), five *V. diabolicus* reference genomes (Vdiab-REF1-REF5), three *Vibrio alginolyticus* reference genomes (Valg-REF1-REF3), and one *Vibrio parahaemolyticus* outgroup (Vpara-OUT). Yellow indicates gene presence (1) and purple indicates absence (0). Genes on the Y-axis are re-ordered by functional role to highlight biological structure in the accessory genome, with blocks corresponding to cell envelope/surface glycan functions (e.g., *pglE, arnB, fdtA, espL_2, legG, pglF_1, wecA*), transport and nutrient uptake (e.g., *btuB_3, group_165, group_1043, group_1083, leuE_3)*, regulation (*marR, vidZ_5)*, translation/protein synthesis (*fmt_2)*, core metabolism (*sdaB_1, aroK_2, vihB)*, and stress/defense-associated functions (*group_604; group_6169* representing an RND-type efflux/defense module). Vertical labels on the left indicate the functional groupings used to arrange the gene order.

Clustering based on accessory gene presence-absence generated relationships distinct from the core-genome phylogeny (Table 5). Heatmap visualization of accessory gene presence-absence profiling of the 13 genomes using the Roary pangenome matrix revealed clear, interpretable patterns once the top 20 selected accessory loci were re-ordered by functional role (Figure 2). Cell envelope and surface-glycan associated genes (e.g., *pglE, arnB, fdtA, espL_2, legG, pglF_1, wecA*) formed a coherent functional block showing non-uniform distribution in genomes, consistent with strain- and species-dependent variation in polysaccharide biosynthesis and outer-surface remodeling. Transport- and nutrient-acquisition genes (including *btuB_3* and accessory transport clusters such as group_165 and group_1043) displayed additional genome-specific patterns, highlighting differences in uptake capabilities among the four isolates, *V. diabolicus* references, and other *Vibrio* species reference genomes. Regulatory and housekeeping-associated genes (*marR, yidZ_5, fmt_2*) and metabolic markers (*sdaB_1, aroK_2*) further separated subsets of genomes, while stress/defense-linked accessory loci (e.g., group_604 and group_6169) were restricted to a smaller subset, suggesting niche- or lineage-specific adaptive elements. Overall, accessory genes revealed pronounced heterogeneity in gene content among isolates, emphasizing the contribution of the flexible genome to intra-species diversity and the function-guided ordering converts the accessory gene matrix into biologically meaningful modules, supporting the conclusion that genome-content differences among the analyzed *Vibrio* lineages are concentrated in surface structure, transport, and adaptive defense functions (Figure 2).

**Table 5:** Comparison of Roary gene clusters between sediment derived and reference *V. diabolicus* genomes.

Group	Total genes present	Core genes	Shell genes	Cloud genes	Core (%)	Shell (%)	Cloud (%)
Sediment isolates (*n* = 4)	4577 ± 212	3453 ± 0	928 ± 48	196 ± 222	75.6 ± 3.4	20.3 ± 1.5	4.1 ± 4.6
Reference genomes (*n* = 5; Vdiab_REF1–REF5)	4703 ± 124	3453 ± 0	963 ± 74	287 ± 100	73.5 ± 1.9	20.5 ± 1.3	6.1 ± 2.0

Comparison of pangenome composition between sediment-derived *Vibrio diabolicus* isolates (n = 4) and reference *V. diabolicus* genomes (n = 5). Values are expressed as mean ± standard deviation in genomes within each group. Percentages indicate the relative contribution of core, shell, and cloud gene clusters to the total gene repertoire, highlighting differences in accessory genome content between environmental isolates and reference strains. Values are mean ± SD across genomes in each group. Core/shell/cloud are defined across the full Roary run; values below are per-genome counts summarized within each group.

### 3.7 Concordance between ANI and core-genome phylogeny

**Figure 3:**
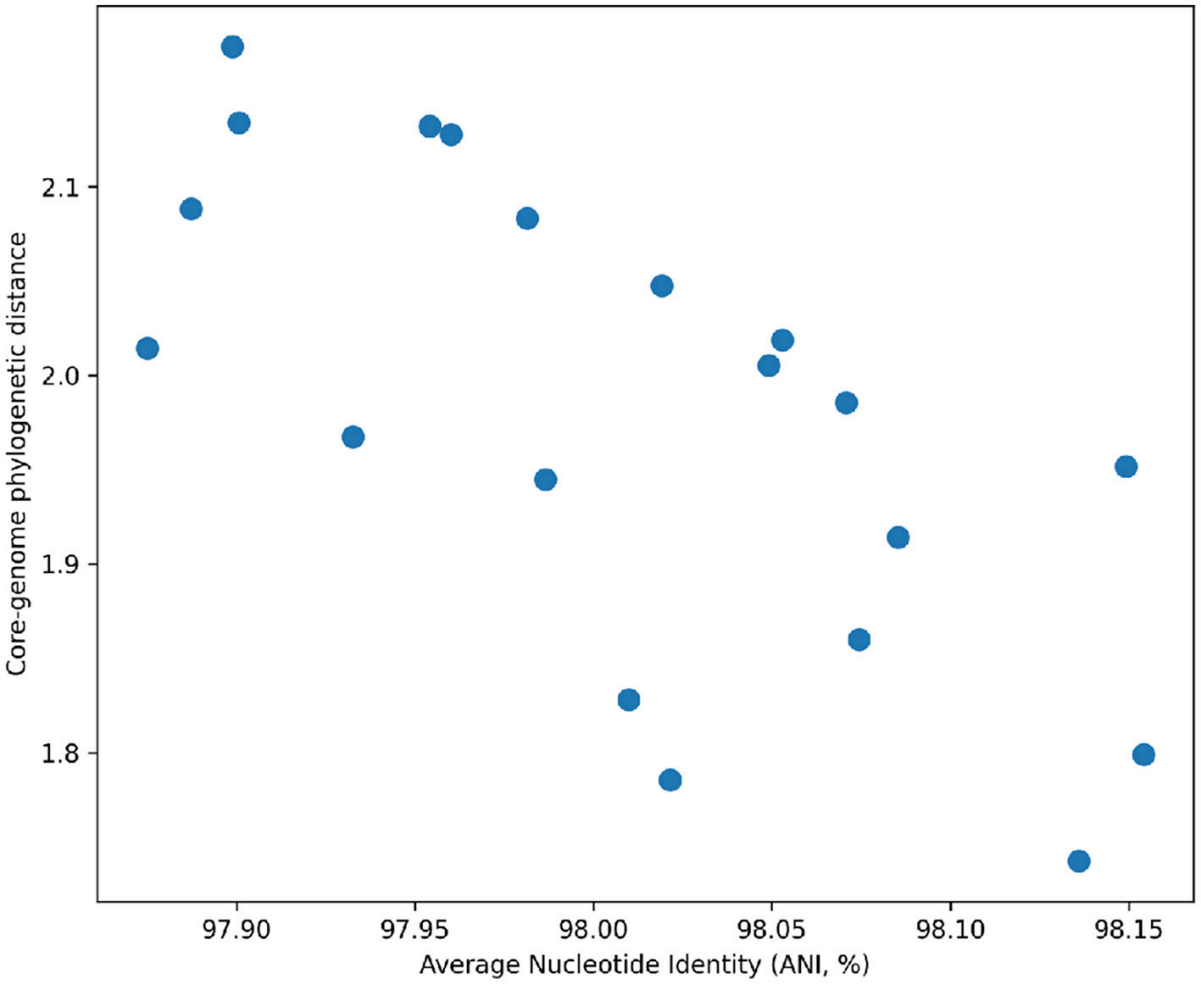
Concordance between ANI and phylogenetic distance. Relationship between pairwise ANI values and core-genome phylogenetic distance among *V. diabolicus* genomes, demonstrating strong agreement between sequence similarity and evolutionary divergence.

Comparison of ANI values with core-genome phylogenetic distances revealed a strong inverse relationship (Table 5; Figure 3), confirming that whole-genome similarity metrics are consistent with evolutionary relationships inferred from conserved genes.

## 4 Discussion

### 4.1 Ecological dominance of *Vibrio diabolicus* in marine sediments

The integration of quantitative culture-based enumeration with genome-resolved analyses provides strong evidence that *V. diabolicus* represents a dominant and ecologically successful *Vibrio* species within marine sediments. The consistently higher CFU values observed for *V*. *diabolicus* relative to other *Vibrio* taxa (Table 1) indicate that this species is not merely present but competitively established in benthic environments. Importantly, this dominance was supported statistically, with log10-transformed CFU counts showing significantly higher abundance of *V. diabolicus* than *V. alginolyticus* (one-way ANOVA, *p* < 0.001). Previous studies have emphasized the importance of *Vibrio spp*. in marine nutrient cycling and biofilm-associated lifestyles (Thompson et al., 2004; Hunt et al., 2008), and the present findings extend this ecological framework specifically to sediment-associate *V*. *diabolicus* populations. Such intra-species ecological success is consistent with broader evidence that marine *Vibrio* populations exhibit substantial genome plasticity shaped by environmental pressures and horizontal gene transfer (Shapiro et al., 2012; Le Roux and Blokesch, 2018). In this context, accessory-genome variation in functions linked to surface structure and nutrient acquisition, traits frequently implicated in sediment persistence and microhabitat colonization, may contribute to the observed ecological dominance of *V. diabolicus* in our site.

### 4.2 General genomic features of *Vibrio diabolicus* isolates

The genomic characteristics observed in the four sediment-derived *Vibrio diabolicus* isolates are highly consistent with previously reported genomes from this species and related members of the Vibrionaceae. For example, the genome of *V. diabolicus* strain 2098 has been reported to be approximately 5.17 Mb within around 4,800 predicted coding sequences, values that closely match the genome size and gene content observed in the isolates analyzed in this study (Xu et al., 2024). Similarly, the type strain *V. diabolicus* CNCM I-1629 possesses a genome of approximately 5.1 Mb, which falls within the same genomic range identified for the isolates L2, L3, VA, and B48 (Raguénès et al., 1997; Le Roux and Blokesch, 2018). More broadly, comparative genomic surveys of the family Vibrionaceae have shown that most *Vibrio* species possess genomes averaging approximately 4.5–5.5 Mb, typically encoding 400–500 protein-coding genes, reflecting a relatively conserved genomic architecture across the genus (Thompson et al., 2004; Le Roux et al., 2015). The genome sizes and coding capacities observed in the present isolates therefore fall well within the expected range for environmental *Vibrio* species. In addition, *Vibrio* genomes are generally organized into two circular chromosomes shaped by recombination and horizontal gene transfer, a genomic organization that contributes to metabolic versatility and environmental adaptation in marine ecosystems (Okada et al., 2005; Le Roux et al., 2016). Taken together, the structural genomic characteristics of isolates L2, L3, VA, and B48 closely mirror those reported for previously sequenced *V. diabolicus* strains and other members of the Harveyi clade, supporting their taxonomic assignment and confirming that the assembled genomes provide a reliable basis for downstream comparative analyses.

### 4.3 Robust species delineation through ANI and phylogenomics

Species-level assignment of the sediment isolates was rigorously supported by both ANI and core-genome phylogenetic analyses. All four isolates exhibited ANI values well above the 95% threshold widely accepted for bacterial species delineation (Goris et al., 2007; Richter and Rosselló-Móra, 2009), while maintaining clear genomic separation from *V*. *alginolyticus* and *V*. *parahaemolyticus* reference genomes (Table 3). Importantly, the core-genome phylogeny mirrored ANI-based relationships (Figures 1–3), reinforcing the concept that ANI and core-gene evolutionary history are highly content metrics for genomic relatedness in *Vibrio*. Such concordance has been repeatedly observed across *Vibrio* comparative genomics studies and is considered a hallmark of robust phylogenomic interference (Thompson et al., 2005; Shapiro et al., 2012). Significantly, the core-genome phylogeny provides robust evidence for the taxonomic placement and evolutionary relationships of the sediment-derived isolates within the *Vibrio diabolicus* lineage. The strong clustering of isolates L2, L3, VA, and B48 with reference *V. diabolicus* genomes confirms that these environmental strains belong to this species and highlights the genomic coherence of the clade (Figure 1). The extremely short branch lengths separating L2 and L3 suggest that these isolates represent closely related environmental variants, potentially originating from the same local population or recent diversifatoin within the sediment microbial community. In contrast, isolates VA and B48 occupy distinct sublineages within the *V. diabolicus* cluster, indicating greater genomic divergence and suggesting the presence of multiple evolutionary lineages of *V. diabolicus* in the sampled sediment environment. Such intra-species diversification is consistent with previous studies showing that marine *Vibrio* populations often exhibit substantial genomic plasticity driven by environmental pressures and horizontal gene transfer (Shapiro et al., 2012; Le Roux and Blokesch, 2018; Turner et al., 2018). The clear separation between *V. diabolicus* and *V. alginolyticus* clades further supports species-level differentiation and validates the phylogenetic framework used in this study. Importantly, the concordance between core-genome phylogeny and accessory gene clustering patterns suggests that strain-level diversification is accompanied by differences in gene content, which may reflect functional adaptations to local environmental conditions and ecological niches in marine sediment habitats.

### 4.4 An open pangenome reflects extensive genomic plasticity

One of the most striking outcomes of this study is the pronounced dominance of accessory genes within the *V*. *diabolicus* pangenome (Table 4). The relatively small core genome, contrasted with a large shell and cloud gene fraction, is characteristic of an open pangenome architecture, as originally conceptualized by (Tettelin et al. 2008). Such pangenome structures are commonly reported for environmentally versatile bacteria and have been documented for multiple *Vibrio* species inhabiting dynamic marine environments (Lukjancenko et al., 2010; Cordero et al., 2012). In the present study, the predominance of cloud genes observed suggests continuous gene acquisition and loss, likely mediated by horizontal gene transfer and selective pressures unique to sedimentary habitats.

### 4.5 Accessory genome drives intra-species differentiation

While core-genome analyses establish species coherence, accessory genome analyses reveal the mechanisms underlying strain-level diversification. Clustering based on accessory gene presence-absence (Figure 1) produced patterns distinct from those observed in the core-genome phylogeny, demonstrating that flexible genome components exert a major influence on genomic relatedness at fine evolutionary scales. The accessory gene heatmap (Figure 2) further illustrates extensive selected strain-specific accessory genes repertoires with known functional annotations, consistent with previous reports that adaptive traits in marine bacteria are frequently encoded within the accessory genome rather than the conserved core (Polz et al., 2013; Shapiro and Polz, 2014). The functional blocks observed in the accessor gene heatmap (Figure 2) especially cell-envelope/surface glycan genes (e.g., *pglE, arnB, fdtA, espL_2, legG, pglF_1, wecA*) together with transport/iron-uptake and defense/efflux loci confirms that much of the intra-species (and within-clade) diversification is concentrated in the flexible genome encoding surface structures, niche-acquisition systems, and defense functions. In the expanded *V. diabolicus* species concept, Turner and colleagues reported “a high degree of individual genome plasticity” and emphasized that the accessory genome contains many defense/virulence-associated and environmentally responsive functions, consistent with lineage-specific gain/loss shaping ecological breadth (Turner et al., 2018). Likewise, broader *Vibrio* pangenome surveys conclude that the flexible gene pool—often carried on mobile elements— drives adaptation and can include virulence and antibiotic-resistance determinants; this provides a clear basis for interpreting the isolated strain-structured presence/absence patterns as adaptive modules rather than random noise (Pérez-Duque et al., 2021). The enrichment of surface-glycan and membrane-associated accessory genes is also consistent with reports in *V. alginolyticus*, where comparative genomics supports an open pangenome and highlights diversification around host-/envirnoment-linked traits while maintains a conserved core (Xue et al., 2022). The presence of putative RND-family efflux components in the accessory set aligns with *Vibrio*-specific literature showing that RND systems are not only “drug pumps” but also contibrbute to stress physiology and colonization-linked regulation, providing a mechanistic basis for why efflux modules often appear as lineage-specific accessories in marine *Vibrio* (Bina and Bina, 2023). Importantly, the coherent blocks of functionally related genes that tend to appear together across genomes mirrors patterns described outside *Vibrio* as a general pangenome principle: in *Escherichia coli*, accessory genes show non-random co-occurrence linked to shared function and mobility, reinforcing the interpretation that the heatmap blocks in Figure 2 likely represent horizontally transferred/maintainted functional cassettes shaped by selection in specific niches (Hall et al., 2021).

### 4.6 Comparative insights within the genus *Vibrio*

In comparison with other *Vibrio* comparative genomics studies, the patterns observed in the present study are broadly consistent yet ecologically informative. Similar open pangenome structures and accessory genome-driven diversification have been described for *V*. *cholerae, V*. *vulnificus*, and *V*. *alginolyticus* (Keymer and Boehm, 2011; Cordero et al., 2012). However, the strong ecological dominance of *V*. *diabolicus* in sediments, coupled with extensive accessory genome diversity, highlights this species as a particularly successful benthic specialist. This combination of dominance and genomic flexibility likely highlights its persistence across spatially and temporally heterogenous sediment environments.

### 4.7 Ecological and evolutionary implications

The concordance between ecological dominance, genomic coherence, and extensive accessory genome diversity suggests that *V*. *diabolicus* occupies a stable yet adaptable ecological niche in marine sediments. The ability to maintain a conserved core genome while simultaneously diversifying through accessory genes may allow rapid response to environmental fluctuations without compromising essential cellular functions. Such genomic strategies are increasingly recognized as central to microbial success in complex ecosystems (Koonin and Wolf, 2008; Polz et al., 2013).

### 4.8 Concluding perspective

By integrating quantitative ecology with genome-resolved comparative analysis, this study provides a comprehensive view of *Vibrio diabolicus* as both an ecologically dominant and genomically dynamic sediment-associated bacterium. The findings establish a robust genomic baseline for understanding *V*. *diabolicus* ecology and evolutions and set the stage for future functional genomics investigations targeting the accessory gene repertoire and its role in sediment adaptation.

## 5 Conclusions

This study provides an integrated ecological and genomic framework for understanding the dominance and diversification of *Vibrio diabolicus* in marine sediment environments. Culture-based enumeration established *V*. *diabolicus* as the most abundant *Vibrio* species in sediment samples, providing a clear ecological rationale for genome resolved investigation. Comparative genomic analyses of four sediment-derived isolates confirmed species-level identity through high ANI values and matching core-genome phylogenetic relationships, demonstrating genomic coherence within the species.

Beyond species confirmation, pangenome analysis revealed that *V*. *diabolicus* possesses an open genome architecture characterized by a limited conserved core and a disproportionately large accessory gene pool. The dominance of shell and cloud genes, together with pronounced strain-specific content, highlights the central role of the flexible genome in driving intra-species diversification and ecological adaptation. Accessory gene clustering and presence-absence patterns further indicate that fine-scale genomic variation among isolates is not captured by core genes alone, emphasizing the importance of accessory genome dynamics in shaping sediment-associated populations.

Collectively, these findings suggest that the ecological success of *V*. *diabolicus* in marine sediments is supported by a dual genomic strategy: conservation of essential core functions coupled with extensive accessory genome plasticity. This combination likely facilitates rapid adaptation to heterogenous and fluctuating sedimentary conditions. The genomic basis established here lays the foundation for future functional genomics studies aimed at elucidating the specific metabolic and ecological roles of accessory genes ultimately advancing our understanding of microbial adaptation and resilience in marine benthic ecosystems.

## Data Availability

The genome assembles generated in this study and all datasets analyzed are available from the corresponding author upon reasonable request. Reference genome sequences were retrieved from the NCBI database. This genome project of *V*. *diabolicus* is indexed in GenBank under the BioProject accession number PRJNA1260419 and the Bio Sample ID SAMN52650543-47. The WGS sequences for *V*. *diabolicus* isolates are available in GenBank under accession numbers JBRZEG000000000 (L2), JBRZEF000000000 (L3), JBRZEE000000000 (VA), and JBRZEI000000000 (B48).
